# The ground reaction force pattern during walking under vestibular-demanding task with/without mastoid vibration: implication for future sensorimotor training in astronauts

**DOI:** 10.3389/fphys.2024.1325513

**Published:** 2024-11-20

**Authors:** Zhuo Wang, Haoyu Xie, Jung Hung Chien

**Affiliations:** ^1^ Rehabilitation Medicine Center and Institute of Rehabilitation Medicine, West China Hospital, Sichuan University, Chengdu, China; ^2^ Key Laboratory of Rehabilitation Medicine in Sichuan Province, West China Hospital, Sichuan University, Chengdu, China; ^3^ Department of Rehabilitation Medicine, the First Affiliated Hospital of Sun Yat-Sen University, Guangzhou, Guangdong, China; ^4^ Department of Health and Rehabilitation Science, College of Allied Health Professions, the University of Nebraska Medical Center, Omaha, NE, United States; ^5^ Independent Researcher, Omaha, NE, United States

**Keywords:** locomotor sensory organization test, gait stability, treadmill-induced perturbations, ground reaction force, vestibular function

## Abstract

**Background:**

The Sensory Organization Test condition 5 (SOT5) assesses an astronaut’s vestibular function pre-/post-spaceflight but has a ceiling effect and mainly evaluates standing balance, neglecting the challenges of walking during space missions. A Locomotor Sensory Organization Test (LSOT) has been developed, mirroring the SOT concept but tailored to assess vestibular function during walking. This study aims to advance current knowledge by examining changes in ground reaction force (GRF) during normal walking (LSOT1) and walking in LSOT5 (vision blocked and treadmill speed varied), both with and without mastoid vibrations.

**Methods:**

Sixty healthy adults were recruited and divided into two groups: one with mastoid vibration and one without. GRF peaks and respective variabilities were analyzed in the vertical (V), anterior-posterior (AP), and medial-lateral (ML) directions during stance cycles. The effects of LSOTs and mastoid vibration on each dependent variable were assessed using Friedman’s two-way analysis of variance by ranks.

**Results:**

The findings revealed that:1) Walking in LSOT5 increased the variabilities of GRFs regardless of the administration of mastoid vibration; 2) the application of mastoid vibration reduced the amplitude of GRF peaks; and 3) walking in LSOT5 while receiving mastoid vibration was the most challenging task compared to all other tasks in this study.

**Conclusion:**

The results indicated that analyzing GRF can detect changes in the strategy of balance control across different sensory-conflicted conditions. The findings could be beneficial for assessing the vestibular function pre- and post-space missions and planning for future sensorimotor training programs aimed at enhancing astronauts’ abilities to navigate unpredictable sensory-conflicted conditions.

## 1 Introduction

In 1969, Astronaut Neil Armstrong initiated his descent down the ladder and articulated, “That is one small step for a man, but one giant leap for mankind.” Subsequently, another astronaut, Dr. Edwin Aldrin, captured his own footprint on the lunar surface (https://nssdc.gsfc.nasa.gov/imgcat/html/object_page/a11_h_40_5878.html, assessed on 20 April 2024). While the footprint registers a depth of approximately one inch, it does not yield insights into the magnitude of the force exerted by Dr. Aldrin during routine ambulation under lunar gravity. On Earth, humans exhibit an extraordinary capacity to perceive gravity, orient themselves within their surroundings, and undertake sensory-motor activities, including walking and maintaining balance amidst challenging environmental conditions, such as darkness, diverse weather phenomena, or surfaces with varying textures such as sand, snow, or wet terrain. The vestibular system serves a pivotal role in detecting head movements, accelerations, and alterations in self-motion relative to gravitational forces (Messina et al., 2021). Additionally, the vestibulo-ocular reflex ensures ocular stability during head movements, thereby maintaining a steady retinal image of the surrounding environment regardless of head motion (Martines et al., 2021). Thus, in a subsequent lunar mission, Dr. Harrison Schmitt, a proficient astronaut, encountered challenges while traversing the lunar terrain, necessitating awkward maneuvers to regain stability (https://www.youtube.com/watch?v=qZBdYp1O2DM, assessed on 20 April 2024). There are a couple of possible reasons that may explain the gait instability experienced by astronauts during moonwalks:1) changes in gravitational forces and 2) alterations in the center of mass location resulting from the spacesuit. For the rationale #1, the gravitational changes on the vestibular system reduce accuracy in detecting self-orientation (Carriot et al., 2021) and generate the inappropriate tilt of body orientation due to the changes in gravity (Reschke et al., 2017), potentially causing falls (e.g., Dr. Schmitt’s example). Moreover, Tays et al. (2021) investigated the vestibular-related balance control in 15 astronauts who had spent approximately 6 months in space upon their return to Earth. Observations indicate that vestibular function takes at least 30 days to fully recover after an extended period of exposure to microgravity, unlike the other two sensory systems (vision and somatosensory systems) (Tays et al., 2021). This highlights the challenge that vestibular function poses when traveling across different planets. However, none of these previous studies have addressed the impact of the spacesuit on gait or stance stability.

The Sensory Organization Test (SOT) has emerged as a pivotal component of clinical assessment for evaluating vestibular function in patients with various vestibular disorders, as extensively documented in prior research (Black et al., 1989; Goebel and Paige, 1989; Hytonen et al., 1989; Mulavara et al., 2013). This test encompasses one baseline and five sensory-conflicted conditions, including scenarios such as 1) eyes open on a fixed surface, 2) eyes closed on a fixed surface, 3) sway-reference vision on a fixed surface, 4) eyes open with sway-reference support, 5) eyes closed with a sway-reference surface, and 6) sway-reference vision with the sway-reference surface. The somatosensory function is discerned by comparing body sway between SOT1 and SOT2, while the visual function is evaluated by comparing sway between SOT1 and SOT4. Moreover, vestibular function has garnered particular interest in numerous studies examining astronauts pre- and post-space missions (Hupfeld et al., 2022; Tays et al., 2021; Shishkin et al., 2023). This evaluation often involves comparing sway between SOT1 and SOT5, employing the principle of simultaneously perturbing the visual and somatosensory systems to indirectly assess vestibular function (Horak, 2007). Horak (2007), in the textbook (Vestibular rehabilitation, third edition, chapter 3. Role of vestibular system in postural control in page 37) describes the SOT5 as: “Vestibular information gives a more accurate estimate of body position and motion under these circumstances (SOT5), and central nervous system (CNS) should rely more heavily on vestibular information for orientation.” Therefore, in following studies, this SOT5 has specifically been used to diagnose the patients with vestibular disorders (Nashner et al., 1982; Black and Nashner, 1984; Black et al., 1988; Shumway-Cook and Horak, 1986) because theoretically the Equilibrium Score should be lower in patients than in healthy controls. Despite its inception approximately 30 years ago, the practice of measuring disparities in sway between SOT1 and SOT5 remains a contemporary method for evaluating vestibular function in astronauts immediately post-spaceflight, as well as for assessing the restoration of balance control in astronauts after extended spaceflight durations (approximately 6 months, Tays et al., 2021; Shishkin et al., 2023). Tays et al. (2021) indicate that microgravity environment resulted in a significant decrease in Equilibrium score in SOT5, indicating a malfunction of the vestibular system after staying microgravity in a period of time. However, a couple of concerns arise regarding this equilibrium score measure: the potential ceiling effect and its sensitivity (Grove et al., 2021). The limit of stability is approximately seven degrees posteriorly and five degrees anteriorly (approximately twelve degrees in range). Participants stepping off from the platform receive an equilibrium score of 0, indicating failure. Within this range, patients with vestibular deficits easily stepped off the platform, leading to only about 50% sensitivity to identify the vestibular deficits using SOT (Di Fabio, 1995). Indeed, these clinical observations of assessing vestibular function through SOT may not be directly applicable to evaluating changes in vestibular function induced by microgravity alterations. It remains reasonable to speculate that the time required for complete adaptation in vestibular function post-space mission could potentially be misinterpreted using SOT, despite its longstanding use in identifying vestibular function in astronauts pre- and post-space missions over the past couple of decades. Most of the aforementioned studies primarily focus on assessing vestibular function pre- and post-space missions while standing but not during walking. Therefore, it is still unknown when these astronauts fully recover their vestibular-related balance during walking. Also, if a walking task is involved in investigating the vestibular function after these astronauts complete their space missions, it typically consists of simple tasks with minimal vestibular demand, such as sit-to-stand, normal walking straight tasks, and obstacle negotiation tasks (Hupfeld et al., 2022). Therefore, it remains uncertain how the disrupted vestibular system (due to exposure to microgravity) affects balance control in astronauts mentioned above when walking under vestibular-demanding tasks, such as navigating dark and unstable surfaces, akin to walking on snowy and slippery ground in the dark of night on Earth.

Expanding upon the foundational principles of the SOT, Chien et al. (2014) devised the Locomotor Sensory Organization Test (LSOT) to assess dynamic balance control (sway) across various sensory challenging conditions. Similar to SOT, LSOT consists of six conditions:

1. Walking normally with full vision on a fixed-speed treadmill.
2. Walking with a blocked vision on a fixed-speed treadmill.
3. Walking with vision-perturbed surroundings on a fixed-speed treadmill.
4. Walking with full vision on a speed-varied treadmill.
5. Walking with blocked vision on a speed-varied treadmill.
6. Walking with vision-perturbed surroundings on a speed-varied treadmill.

For LSOT 5, participants walked on the treadmill with perturbations while wearing blackout goggles covered with a layer of 5% car-tinting vinyl, effectively blocking peripheral vision. A small amount of light was permitted to penetrate the goggles to simulate reduced lighting conditions, with light intensity reduced from approximately 150 lx (typical office lighting) to approximately 0.7 lx (comparable to the full Moon on a dark street without streetlights). Wearing this specific goggle reduces the visibility and further forces thirty healthy young individuals to increase the level of active control by increasing the range of heel placements on treadmill between steps (Ren et al., 2022). This result indicates that the increase in active control of heel placement is attributed to a compensatory strategy that utilizes proprioceptive, somatosensory, and vestibular inputs to maintain dynamic balance in conditions of restricted vision. Furthermore, walking or standing in such limited visual environments has been shown to improve balance in patients with Parkinson’s Disease (Tramontano et al., 2016; Bonnì et al., 2019) and in individuals with unilateral lower-limb amputations (Vrieling et al., 2008).

However, it could be argued that the design of LSOT4-6 may have a potential drawback, as the changes in balance control during gait could be solely attributed to alterations in sudden acceleration by the treadmill rather than sensory conflicts. To address this, in the LSOT5 condition, the mean speed within treadmill-induced perturbations is set to 99.2% of the preferred walking speed, closely mirroring the preferred walking speed observed in LSOT1 (Wang et al., 2024). This method allows for the averaging out of any changes solely induced by sudden accelerations in LSOT5 compared to LSOT1. Wang et al. (2024) further observe that there is no significant difference in step length, which is highly related to treadmill speed because the differences in step length were averaged out. However, the margin of stability is significantly smaller in LSOT5 than LSOT1, indicating that walking in LSOT5 indeed triggers the sensory reweighting process (Wang et al., 2024). However, it can also be debated whether the vestibular system specifically plays a role in controlling balance in LSOT5. This argument can be explored by implementing vestibular stimulation through bilateral mastoid vibration (Lin et al., 2021; Sun et al., 2023). If walking in LSOT5 specifically involves balance control by the vestibular system, mastoid vibration would alter the balance control compared to conditions without mastoid vibration during walking. Based on Chien et al.‘s findings, walking in LSOT5 with mastoid vibrations (MV), whether unilaterally or bilaterally applied, markedly increases the variability of the net center of pressure sway in both young adults (Chien et al., 2016) and older adults (Chien et al., 2017) compared to walking in LSOT5 without mastoid vibration. In fact, it has been proposed that any alterations in body acceleration prompt a reliance on the vestibular system (Wibble et al., 2020). Therefore, it can be explained that when walking in LSOT5, the Central Nervous System (CNS) may prioritize the vestibular system over the other two sensory systems to maintain balance.

In order to measure balance control during walking, ground reaction force (GRF) is commonly measured in patients with strokes (Chen et al., 2007; Hsiao et al., 2016; Kim and Eng, 2003), Parkinson’s disease (Alam et al., 2017), peripheral arterial disease (Scott-Pandorf et al., 2007), and in those with disturbed unilateral vestibular systems (Magnani et al., 2021). As a result of stepping on the force plates while walking, the ground generated an equal and opposite reaction force for each foot, allowing the identification of the force applied to the ground and acceleration-related data to be obtained. During the initial single support phase, the center of mass was transferred from the lowest to its highest location, leading to a peak in the vertical direction (V1). Also, the second peak (V2) occurred during the late single support phase to slow and control the downward movement of the center of mass. It should be noted that the first peak in the anterior-posterior direction (AP1) represented deceleration due to posterior shear force whereas the second peak demonstrated pushing off, which propelled the body forward. Also, when the heel strikes initially, there is a lateral thrust in the medial-lateral direction (ML). In the final push-off stage, a small lateral force was observed after the body moved over the stance limb. Specifically, Magnani et al. (2021) suggested that the vestibular system was critical to the control of GRFs in the ML direction while unilateral vestibular function was disrupted. Interestingly, the alterations in GRFs during walking under vestibular-demanding tasks, where both vestibular systems are perturbed bilaterally simultaneously, remain undiscovered. Understanding this knowledge gap could establish fundamental concepts of ground reaction force (GRF) applied under such conditions, thereby informing future space missions, such as walking on dark and quicksand surfaces on Mars. Since spatial-temporal gait parameters (Chien et al., 2014), the net center of pressure (Chien et al., 2016; Chien et al., 2017), heel placement (Hu and Chien, 2021), and margin of stability (Wang et al., 2024) have been investigated in LSOT1 and LSOT5, this study aimed to expand upon the existing knowledge by examining the changes in GRF while walking in LSOT5 (vestibular-demanding task) with and without MV (vestibular disruption).

A large, well-equipped facility such as NASA has the capability to measure changes in vestibular-related balance control in response to alterations in gravity. However, the costs associated with such measurements in vestibular-related balance control may not be justified in a typical biomechanical laboratory. Nonetheless, a viable and cost-effective method exists for assessing vestibular-related balance control in vestibular-perturbed and vestibular-demanding environments through the utilization of vestibular stimulation (mastoid process). Specifically, the application of mastoid stimulation has been demonstrated to increase the Center of Gravity (CoG) sway area in both young and older adults during standing (Lin et al., 2022; Zhang et al., 2024), as well as the variability of the net Center of Pressure sway area in both young and older adults (Chien et al., 2016; Chien et al., 2017). These findings affirm the feasibility of employing mastoid vibrations to disrupt vestibular function. Consequently, this study aimed to utilize bilateral MV to simulate scenarios wherein astronauts (serving as healthy controls in the present study) walk with an unreliable vestibular system due to fluctuations in gravity levels during vestibular-demanding tasks.

This study was supported by NASA as a pilot investigation focused on identifying force shifts during walking, particularly in relation to the vestibular system under sensory-conflicted conditions, to inform future research. To the best of our knowledge, this study is the first to examine how GRF patterns change when walking in a vestibular demanding environment, both with and without vestibular disruption. Given the uncertainties involved, this study aimed to explore GRF patterns and their respective variability across all three directions. Hence, the aims of this study were to determine 1) whether walking in vestibular-demanding environment (LSOT5) altered the GRF patterns compared to walking normally (LSOT1); 2) when the vestibular system was disrupted bilaterally, what changes in GRF patterns would be observed in normal walking (LSOT1) and in vestibular-demanding conditions? Enhanced comprehension of GRFs could assist physicians and astronauts in discerning the role of vestibular function in controlling force shifts during walking in various gravity levels and vestibular-demanding environments. This study hypothesized that 1) walking in LSOT5 decreased the GRFs and increased the GRF variabilities; 2) applying MV decreased the GRFs and increased the GRF variabilities; and 3) LSOT5 with bilateral MV would be the most challenging task compared to other conditions, indicating that LSOT5 can be used to identify the deteriorations in vestibular system.

## 2 Materials and methods

### 2.1 Participants

In this investigation, a cohort of sixty young adults participated. These individuals were divided into two distinct groups: 1) the no mastoid vibration group (NoMV) and 2) the mastoid vibration group (MV). The NoMV group comprised 15 males and 15 females, with an average participant age of 22.9 ± 2.11 years (range: 8), an average height of 1.70 ± 0.07 (range: 0.28) m, and an average weight of 66.59 ± 8.52 (range: 32) kg, exhibiting a preferred walking speed of 1.48 ± 0.22 (range: 0.8) m/s. Correspondingly, the MV group consisted of 16 males and 14 females, with comparable demographic characteristics: an average age of 24.3 ± 2.89 (range: 10) years, an average height of 1.71 ± 0.08 (range: 0.31) m, an average weight of 67.87 ± 7.5 (range: 30) kg, and a preferred walking speed of 1.52 ± 0.27 (range: 1.1) m/s. It has been shown that the walking speed may affect the perception of vestibular function (Anson et al., 2019). Therefore, this study attempted to match the age, height, weight and preferred walking speeds between groups as close as possible to limit the effect of confounding factors (Table 1). Importantly, none of the participants reported any ankle, knee, or hip injuries that could potentially influence their gait patterns by self-reporting. Moreover, they had no history of falls in the preceding year and exhibited no deficits in visual, somatosensory, or vestibular functions by self-reporting. Participants were required to achieve a Dizziness Handicap Inventory score of 0, indicating the absence of vestibular impairments. Otherwise, the participant would have been excluded from the study. It should be noted that this score on the Dizziness Handicap Inventory was for inclusive criteria and this score was not the dependent variable in the present study. Ethical considerations were rigorously observed throughout the study, as evidenced by the approval of the University of Nebraska Medical Center Institutional Review Board (IRB# 340-10-FB). Prior to data collection, each participant voluntarily provided informed consent by signing a consent form on the day of the experiment.

**TABLE 1:** Participants’ information.

	Age (yrs)		Height (m)		Weight (kg)		Walking speed (m/s)	
	Group#1	Group#2	Group#1	Group#2	Group#1	Group#2	Group#1	Group#2
	24	30	1.68	1.66	57	51	1.2	1.2
	23	23	1.63	1.66	75	75	1.3	1.3
	22	20	1.82	1.83	75	70	1.5	1.5
	22	22	1.77	1.71	49	51	1.3	1.3
	27	28	1.67	1.69	45	75	1.5	1.5
	23	23	1.73	1.72	54	54	1	1.1
	25	25	1.76	1.78	74	71	1.8	1.9
	22	22	1.65	1.65	70	74	1.5	1.4
	24	23	1.78	1.79	73	75	1.7	1.7
	25	24	1.79	1.78	51	57	1.7	1.7
	26	30	1.56	1.59	76	75	1.6	1.6
	26	27	1.68	1.83	73	81	1.7	1.8
	21	28	1.68	1.71	77	74	1.7	1.7
	21	20	1.56	1.52	76	75	1.4	1.4
	20	26	1.74	1.8	67	66	1.3	1.3
	23	23	1.7	1.64	67	66	1.8	1.8
	20	20	1.72	1.7	68	67	1.5	1.5
	20	22	1.69	1.67	68	65	1.6	1.6
	22	23	1.68	1.68	63	66	1.1	0.8
	22	25	1.73	1.76	65	66	1.8	1.9
	22	23	1.76	1.8	70	72	1.3	1.4
	25	26	1.75	1.71	70	66	1.3	1.5
	22	25	1.72	1.72	57	61	1.7	1.8
	22	24	1.54	1.64	69	72	1.7	1.9
	25	27	1.76	1.82	66	66	1.3	1.2
	23	27	1.72	1.77	70	72	1.5	1.6
	22	22	1.61	1.66	76	75	1.7	1.9
	21	21	1.73	1.72	64	66	1.4	1.5
	21	21	1.71	1.7	64	61	1.3	1.4
	28	28	1.69	1.61	69	71	1.3	1.4
Avg	22.97	24.27	1.70	1.71	66.60	67.87	1.48	1.52
Std	2.11	2.90	0.07	0.08	8.52	7.51	0.22	0.27

For the purpose of estimating the sample size, two sources were used: 1) prior studies and 2) power estimation using G*Power (URL: http://www.gpower.hhu.de/). From the Chien et al.‘s study (2016), which investigating the net center of pressure area variability calculated by force plate under different LSOT x MV conditions in twenty healthy young individuals, the partial eta squared values were 0.982 for the effect of LSOT effect, 0.913 for the effect of MV, and 0.388 for the interaction between the effect of LSOT and the effect of MV. These partial eta squared values indicated the large effect size (>0.138) according to the Cohen’s textbook (1988). Also, from Lu et al., study (2022), which investigating the different MV effect on margin of stability (MOS) in twenty healthy young adults, the partial eta squared values were 0.755 for MOS in the anterior-posterior and 0.695 for MOS in the medial-lateral directions, indicating the large effect size. A power estimation using G * Power 3.1 was used to estimate the statistical power. The MANOVA for statistical test and *a priori*: compute required sample size–given alpha, power, and effect size for type of power analysis, and the effect size f(v) = 0.565 ∼ partial eta squared value = 0.059 were selected and the result showed that total sample size = 43 (22 for each group) could reach the power of 95%. Also, when 40 participants were recruited (20 per each group by aforementioned studies), the means (LSOT1: 0.32, LSOT5: 0.34, LSOT1MV: 0.29, LSOT5MV: 0.25) and standard deviations (LSOT1: 0.06, LSOT5: 0.05, LSOT1MV: 0.05, LSOT5MV: 0.05) of first peak of GRF in the anterior-posterior direction were used to calculate the power using G * Power (f(v) = 0.425) and the result indicated that recruiting a total sample size of 60 participants (30 per each group) can reach 90% of power for interpreting the outcomes. Thus, in the current study, recruiting 30 participants in each group should have sufficient power to interpret the results in the current study.

### 2.2 Experimental setup

**FIGURE 1:**
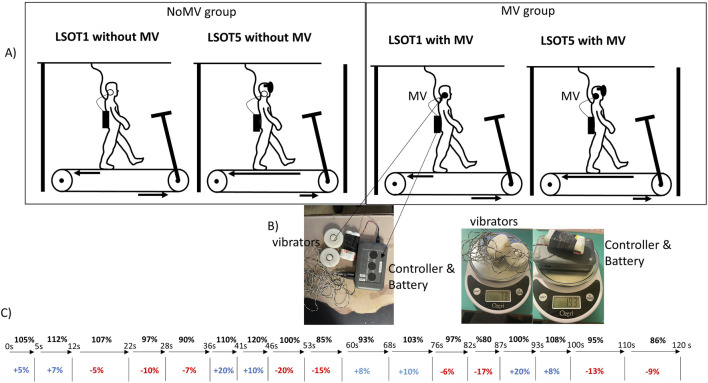
**(A)** The LSOT (locomotor sensory organization test) diagram. LSOT 1: walking at a constant and comfortable speed on the treadmill with full vision. LSOT5: participants walked on the treadmill perturbation and wore blackout goggles with a layer of 5% car-tinting vinyl. **(B)** the diagram of mastoid vibration to stimulate the mastoid process and weight of MV equipment, **(C)** The sequence of 17 blocks of treadmill perturbations.

In this study, a treadmill equipped with two force plates (FIT5, Bertec Corp., Columbus, OH, USA) beneath two belts, one for each leg, was utilized to measure ground reaction force (GRF) during walking in the anterior-posterior (AP), medial-lateral (ML), and vertical (V) directions. Each leg had its independent force plate, and the sampling rate for ground reaction forces was 300 Hz. The Locomotor Sensory Organization Test (LSOT) comprised six conditions, as previously described in Chien et al.'s research on locomotor sensory organization test (Chien et al., 2014; Chien et al., 2016; Chien et al., 2017). In LSOT 5, the treadmill speed changed every 5–10 s to ensure at least five strides between each alteration in speed. This design was based on prior studies, which suggested that allowing at least four to five strides (around 5 s) between treadmill perturbations could mitigate the risk of falling and facilitate recovery from the perturbations (Forner Cordero et al., 2003). A maximum of 10 s (approximately 8–10 strides) between speed changes was implemented to create a continuous alteration in walking speeds for participants. Treadmill-induced perturbations were designed as follows (refer to Figure 1.): Step #1) time blocks were generated continuously and randomly until the sum of these values reached 120 s; Step #2) preferred walking speed (PWS) blocks were generated within a range of −20%–20% (positive values indicating acceleration, negative values indicating deceleration). This value was assigned to the time blocks and added to the previously generated values. The range of walking speed (80%–120% of PWS) was selected to avoid significant changes in gait patterns. In this study, 17-time interval blocks were established, and the speed alterations are depicted in Figure 1 (Chien et al., 2014; Chien et al., 2016; Chien et al., 2017; Hu and Chien, 2021; Wang et al., 2024). The sudden acceleration of the treadmill belt was set at 8 m/s^2 to induce the perturbations (Song et al., 2021), and these speed alterations were controlled by a customized visual basic script (Microsoft, Redmond, USA). Only LSOT one and five were utilized in this study to align with its objectives, focusing solely on investigating the vestibular system. For LSOT 5, participants walked on the treadmill with perturbations while wearing blackout goggles covered with 5% car-tinting vinyl, effectively blocking peripheral vision. A small amount of light was permitted to penetrate the goggles to simulate reduced lighting conditions, with light intensity reduced from approximately 150 lx (typical office lighting) to approximately 0.7 lx (comparable to the full Moon on a dark street without streetlights). Light intensities were measured using a light meter (Dr. Meter, support@drmeter.com) inside the goggles, and room light intensities were monitored between trials to ensure consistency throughout data collection. For the Mastoid Vibration (MV) group, the bilateral mastoid vibrations were generated by two electromechanical vibrotactile transducers (EMS2 tactors; Engineering Acoustics, Casselberry, FL, USA; see Figure 1). These transducers were affixed inside a customized swim cap using double-sided adhesive strips and could be adjusted to position them on the mastoid processes bilaterally. Designed for mounting with a cushion, they could produce high displacement levels, enabling the vibration to be easily sensed even through layers of padding. The controller and battery weighted 193 g and two vibrators weighted 73g, so total weight of this MV equipment was 266 g (Figure 1). A frequency of 100 Hz was chosen for bilateral mastoid process stimulation as it has been demonstrated to trigger nystagmus and necessitate compensatory responses from the vestibular system in healthy young adults (Perez, 2003), patients with vestibular neuritis (Nuti and Mandalà, 2005), and patients with otosclerosis (Manzari et al., 2008). The amplitude of supra-threshold vibrations was set at 130% of the amplitude perceivable by the participants (Lu et al., 2022). The frequency and amplitude of the mastoid vibrations were controlled by software (TAction Creator; Engineering Acoustics, Casselberry, FL, USA) by transmitting the designed signal from the laptop to the controller via Bluetooth technology. The minimum perceived amplitude was determined by adjusting the vibration amplitude through the TAction Creator commercial software until participants could perceive it while standing. The vibrations were administered to participants on both mastoid processes simultaneously. The vibration activation followed an impulse-type pattern, with a 0.5 s activation period and a 0.5 s deactivation period (Lu et al., 2022). The rationale for using this impulse-type vibration was to mitigate the saturation of the vestibular sensation (Chien et al., 2016).

### 2.3 Experimental protocol

After participants voluntarily signed the informed consent, PWS needed to be obtained. For both groups, the experimenters increased the treadmill speed to 0.8 m/s and instructed participants to step on the belt. After 20 s, participants were asked whether this speed was comfortable, like walking around the neighborhood. The speed was increased or decreased by 0.1 m/s based on participants’ responses by experimenters. This procedure was performed repeatedly until the participants confirmed the PWS. Once the PWS was identified, the participants walked on the treadmill for 5 minutes to familiarize themselves with treadmill walking. After familiarization with treadmill walking, participants took a 2-min mandatory rest. Then, participants were randomly assigned into two groups: NoMV and MV groups. Next, two conditions (LSOT1 and LSOT5) will be assigned randomly to these participants. For the NoMV group, participants walked in LSOT1 and LSOT5 without any mastoid vibration. Conversely, for the MV group, participants walked in LSOT1 and LSOT5 with bilateral mastoid vibrations. It should be noted that both MV and control groups wear this MV equipment (two transducers were attached on two sides of mastoid process, and a control attached on around location of sacrum). Also, the mixed experimental design (within group: LSOT1 vs. LSOT5; between groups: the effect of mastoid vibration) was implemented according to previous published study (Wang et al., 2024). Wang et al. (2024) investigates the margin of stability when walking on similar LSOT conditions (LSOT1, LSOT4, and LSOT5) similar to the present study by using a single group and find an apparent limitation–the learning effect between similar conditions although a 2-min mandatory rest between conditions is provided to participants. They wrote “the limitation was whether a 2-min rest was enough to eliminate the learning effect. This present study did not provide sufficient, direct evidence to support this claim.” In the current study, if the same participants were assigned to walk two LSOT5, the potential learning effect may be inevitable. Thus, in this current study, a mixed experimental was used. Also, each participant only experience LSOT5 one time. All participants wore the MV device, but the MV only was applied in the MV group. In LSOT5, the sequence of blocks was presented in the same order to each participant to ensure that the outcomes remained comparable both within and between groups. Also, between conditions, a 2-min mandatory rest was assigned to participants to catch their breath. Each LSOT condition lasted 2 minutes. Thus, two 2-min LSOT conditions (one LSOT1 and one LSOT5) were assigned to each participant. At the end of each trial, the participants were instructed to sit on a chair with handles. They were asked if they felt uncomfortable sensations like nausea, vomiting, or dizziness. If participants experienced any discomfort, the experiment was immediately terminated. Also, each participant was asked to verbally describe their experience while walking after each condition.

### 2.4 Data analysis

**FIGURE 2:**
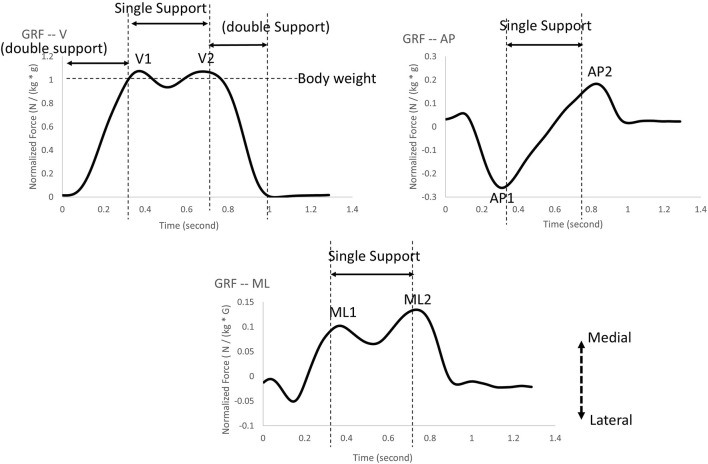
The ground reaction force peaks were selected in the present study: V1 and V2 represent ground reaction force peaks in the vertical direction; AP1 and AP2 represent ground reaction force peaks in the anterior-posterior direction; ML1 and ML2 represent ground reaction force peaks in the medial-lateral direction.

The GRF were analyzed along the vertical (V), anterior-posterior (AP), and medial-lateral (ML) directions. Initially, the raw GRF data from the instrumented treadmill were filtered using a fourth-order low-pass Butterworth filter with a 10 Hz cut-off frequency (McCaw et al., 2013). The GRF components in each direction were reported for peaks V1, AP1, ML1, and V2, AP2, and ML2 (Figure 2. All GRFs were normalized with respect to each participant’s body weight (McCaw et al., 2013). The GRF was utilized to discern a crucial gait event—initial heel contact. The initial heel contact was determined as the instant when the vertical component of the ground reaction force exceeded 10 N and was sustained for 40 ms (Chien et al., 2014). A stance cycle represented the duration between two consecutive initial heel contacts for each leg. The GRF variability was defined as the coefficient of variation of each dependent variable within the gait cycles observed over a period of 2 minutes for each trial.

### 2.5 Statistical analysis

The dizziness handicap score was not included in the statistical analysis here because participants with a score greater than zero were excluded from the study.

A Shapiro-Wilk normality test with an alpha value of 0.05 was used to evaluate the normality for each dependent variable and participants’ information. All data were analyzed using SPSS (26.0).

• If the participants’ information were normally distributed, the independent t-test was used; otherwise, the Mann-Whitney Test was used.
•If the data were normally distributed, a mixed two-way repeated measure ANOVA (2 LSOT conditions x mastoid vibration) was used to investigate the condition effect and mastoid vibration effect as well as the interaction between these two effects. If a significant interaction was found, pairwise comparisons were corrected by the Bonferroni method. An independent t-test was used to compare between groups and a pair t-test was used to compare means within conditions.
•If the data were not normally distributed, Friedman’s two-way analysis of variance by ranks was used. If the Friedman test showed significance the Mann-Whitney Test was used to compare means between groups (e.g., LSOT1 vs. LSOT1MV) and the Wilcoxon Signed Ranks Test was used to compare means from a same group in different conditions (LSOT1 and LOST5).
• It should be noted that the Bonferroni corrections were applied in all pairwise comparisons (6 * 12 = 72, as there were 12 variables); therefore, the significant level was 0.05/72 = 0.0007. The alpha value needed to be smaller than 0.0007 to be significant.

The effect size was calculated using partial eta squared values for normal distribution data (small effect: 0.01, medium effect: 0.06, large effect: 0.14) and using Kendalls W values for non-normal distribution data (small effect: 0.1, medium effect: 0.3, large effect: 0.5). All criteria for identifying the effect size using Cohen’s interpretation guidelines (Cohen, 1988).

## 3 Results

### 3.1 Participants’ information between two groups (NoMV and MV groups)

There were no significant differences in age (*p* = 0.072), height (*p* = 0.576), weight (*p* = 0.750), and preferred walking speed (*p* = 0.425). More details are shown in Table 1.

### 3.2 Normalized test

The Shapiro-Wilk normality test showed that.

• Normally distribution in AP1, ML1, ML2.
• Non-Normally distribution in V1, V1V, V2, V2V, AP1V, AP2, AP2V, ML1V, ML2V.

### 3.3 Effects of LSOT conditions and bilateral mastoid vibrations

• A two-way mixed ANOVA repeated measure was used to investigate whether AP1, ML1, and ML2 differed in different LSOT conditions with/without bilateral MV. A significant interaction was found in aforementioned variables (AP1: F_1,58_ = 22.905, *p* < 0.0001; ML1: F_1,58_ = 6.588, *p* = 0.013; ML2: F_1,58_ = 11.544, *p* = 0.001).
• Friedman test was conducted to determine whether each dependent variable differed in different LSOT conditions with/without bilateral mastoid vibrations. A significant difference was found in V1 (χ^2^ (3) = 72.6, *p* < 0.001), VV1 (χ^2^ (3) = 40.92, *p* < 0.001), V2 (χ^2^ (3) = 52.56, *p* < 0.001), VV2 (χ^2^ (3) = 62.96, *p* < 0.001), APV1 (χ^2^ (3) = 58.08, *p* < 0.001), AP2 (χ^2^ (3) = 34.6, *p* < 0.001), AP2V (χ^2^ (3) = 64.52, *p* < 0.001), ML1V (χ^2^ (3) = 34.68, *p* < 0.001), and ML2V (χ^2^ (3) = 35.08, *p* < 0.001).

**FIGURE 3:**
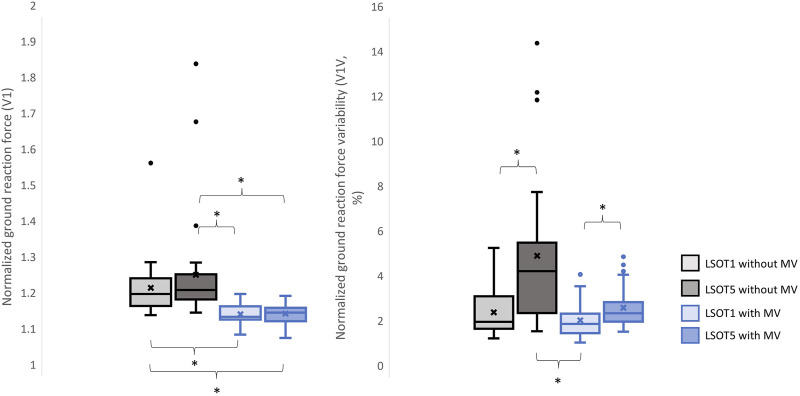
The normalized GRF V1 and respective variabilities, V1V, *: *p* < 0.0007.

**FIGURE 4:**
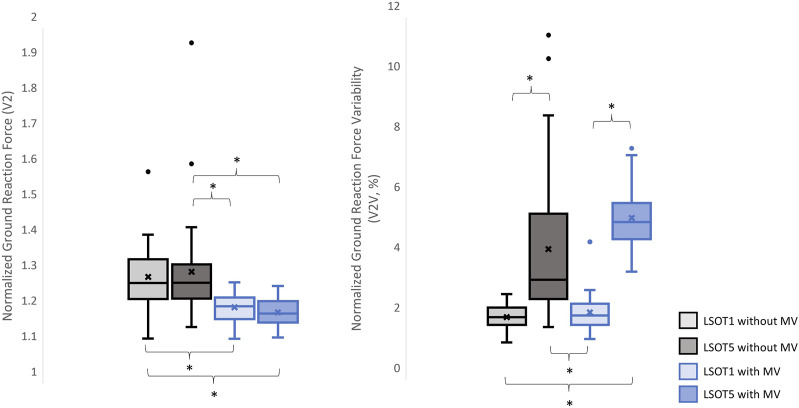
The normalized GRF V2 and respective variabilities, V2V, *: *p* < 0.0007.

**FIGURE 5:**
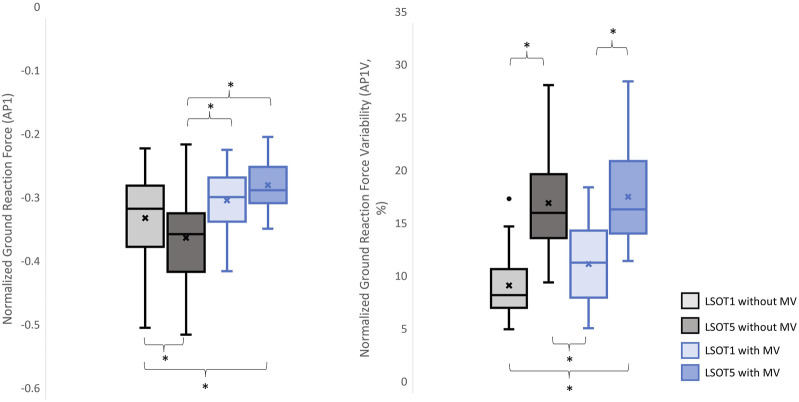
The normalized GRF AP1 and respective variabilities, AP1V, *: *p* < 0.0007.

**FIGURE 6:**
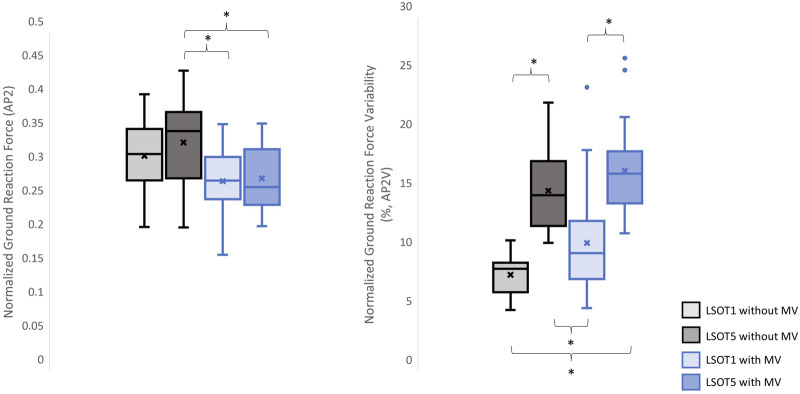
The normalized GRF AP2 and respective variabilities, AP2V, *: *p* < 0.0007.

**FIGURE 7:**
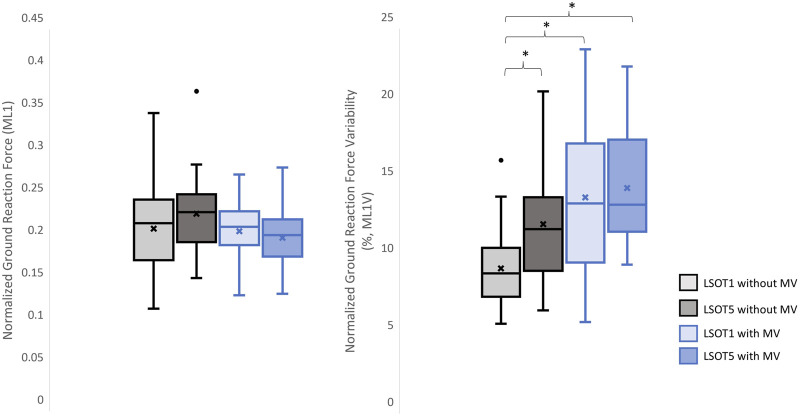
The normalized GRF ML1 and respective variabilities, ML1V, *: *p* < 0.0007.

**FIGURE 8:**
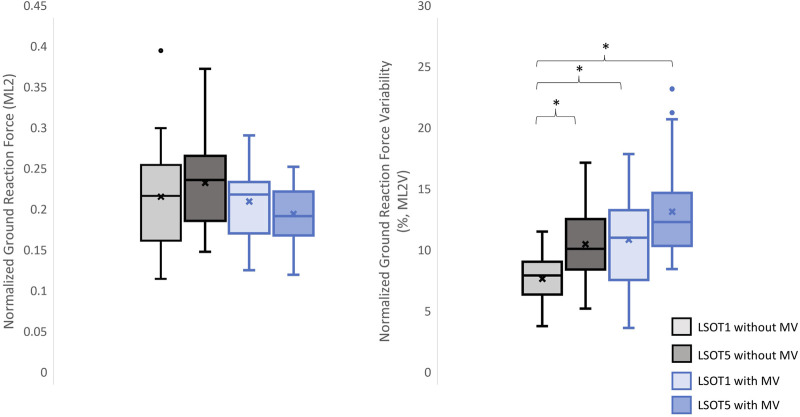
The normalized GRF ML2 and respective variabilities, ML2V, *: *p* < 0.0007.

The means, medians, and standard deviations are shown in Table 2. The pairwise comparisons are shown in Table 3, Figures 3–8.

**TABLE 2:** Means, Median, and Standard deviation of each dependent variables.

V1	LSOT1	LSOT5	LSOT1_MV	LSOT5_MV
Mean	1.25	1.28	1.17	1.17
Median	1.23	1.24	1.17	1.18
Standard Deviation	0.08	0.15	0.02	0.03

V1V	LSOT1	LSOT5	LSOT1_MV	LSOT5_MV
Mean	2.90	5.42	2.55	3.10
Median	2.47	4.73	2.38	2.86
Standard Deviation	1.02	3.45	0.83	0.91

V2	LSOT1	LSOT5	LSOT1_MV	LSOT5_MV
Mean	1.30	1.31	1.21	1.20
Median	1.28	1.28	1.22	1.20
Standard Deviation	0.09	0.15	0.04	0.04

V2V	LSOT1	LSOT5	LSOT1_MV	LSOT5_MV
Mean	2.07	4.33	2.23	5.37
Median	2.07	3.31	2.13	5.23
Standard Deviation	0.41	2.55	0.62	1.04

AP1	LSOT1	LSOT5	LSOT1_MV	LSOT5_MV
Mean	−0.31	−0.35	−0.29	−0.26
Median	−0.30	−0.34	−0.28	−0.27
Standard Deviation	0.07	0.07	0.05	0.04

AP1V	LSOT1	LSOT5	LSOT1_MV	LSOT5_MV
Mean	10.19	17.98	12.22	18.58
Median	9.27	17.04	12.35	17.38
Standard Deviation	3.00	4.50	3.66	4.46

AP2	LSOT1	LSOT5	LSOT1_MV	LSOT5_MV
Mean	0.32	0.34	0.28	0.28
Median	0.32	0.35	0.28	0.27
Standard Deviation	0.05	0.06	0.05	0.04
Mean	8.21	15.32	10.89	17.03
Median	8.71	14.94	10.04	16.77
Standard Deviation	1.67	3.23	4.17	3.61

ML1	LSOT1	LSOT5	LSOT1_MV	LSOT5_MV
Mean	0.22	0.23	0.21	0.20
Median	0.22	0.23	0.22	0.21
Standard Deviation	0.05	0.04	0.03	0.03

ML1V	LSOT1	LSOT5	LSOT1_MV	LSOT5_MV
Mean	9.44	12.32	14.05	14.66
Median	9.11	11.98	13.66	13.57
Standard Deviation	2.49	3.77	4.69	3.95

ML2	LSOT1	LSOT5	LSOT1_MV	LSOT5_MV
Mean	0.23	0.25	0.23	0.21
Median	0.23	0.25	0.23	0.21
Standard Deviation	0.06	0.05	0.04	0.03

ML2V	LSOT1	LSOT5	LSOT1_MV	LSOT5_MV
Mean	8.67	11.50	11.88	14.17
Median	8.94	11.13	12.03	13.31
Standard Deviation	1.84	3.06	3.81	3.86

**TABLE 3:** Statistical Analysis, the bold fonts indicated the significance. The level of significance was 0.0007 for pairwise comparisons. S: significant. NS: not significant.

V1 (Friedman test, *p* < 0.001)				
	LSOT1	LSOT5	LSOT1_MV	LSOT5_MV
Normal	X	*p* = 0.039 (NS)	**p < 0.0001 (S)**	**p < 0.0001 (S)**
LSOT5		X	**p < 0.0001 (S)**	**p < 0.0001 (S)**
Normal_MV			X	*p* = 0.943
LSOT5_MV				X

V1V (Friedman Test, *p* < 0.001)				
	LSOT1	LSOT5	LSOT1_MV	LSOT5_MV
Normal	X	**p < 0.0001 (S)**	*p* = 0.179	*p* = 0.152
LSOT5		X	*p* = 0.0011 (NS)	**p < 0.0001 (S)**
Normal_MV			X	**p < 0.0001 (S)**
LSOT5_MV				X

V2 (Friedman Test, *p* < 0.001)				
	LSOT1	LSOT5	LSOT1_MV	LSOT5_MV
Normal	X	*p* = 0.845	**p < 0.0001 (S)**	**p < 0.0001 (S)**
LSOT5		X	**p < 0.0001 (S)**	**p < 0.0001 (S)**
Normal_MV			X	*p* = 0.026 (NS)
LSOT5_MV				X

V2V (Friedman Test, *p* < 0.001)				
	LSOT1	LSOT5	LSOT1_MV	LSOT5_MV
Normal	X	**p < 0.0001 (S)**	*p* = 0.478	**p < 0.0001 (S)**
LSOT5		X	**p < 0.0001 (S)**	*p* = 0.0014 (NS)
Normal_MV			X	**p < 0.0001 (S)**
LSOT5_MV				X
Normal	X	**p = 0.0001(S)**	*p* = 0.073	**p = 0.0006(S)**
LSOT5		X	**p = 0.0002(S)**	**p < 0.0001(S)**
Normal_MV			X	*p* = 0.014 (NS)
LSOT5_MV				X

AP1V (Friedman Test, *p* < 0.001)				
	LSOT1	LSOT5	LSOT1_MV	LSOT5_MV
Normal	X	**p = 0.0001(S)**	*p* = 0.023 (NS)	**p < 0.0001(S)**
LSOT5		X	**p < 0.0001(S)**	*p* = 0.605
Normal_MV			X	**p = 0.0001(S)**
LSOT5_MV				X

AP2 (Friedman Test, *p* < 0.001)				
	LSOT1	LSOT5	LSOT1_MV	LSOT5_MV
Normal	X	*p* = 0.041 (NS)	*p* = 0.006 (NS)	*p* = 0.007 (NS)
LSOT5		X	**p < 0.0001 (S)**	**p = 0.0005 (S)**
Normal_MV			X	*p* = 0.673
LSOT5_MV				X

AP2V (Friedman Test, *p* < 0.001)				
	LSOT1	LSOT5	LSOT1_MV	LSOT5_MV
Normal	X	**p < 0.0001 (S)**	*p* = 0.006 (NS)	**p < 0.0001 (S)**
LSOT5		X	**p < 0.0001 (S)**	*p* = 0.069 (NS)
Normal_MV			X	**p < 0.0001 (S)**
LSOT5_MV				X
Normal	X	*p* = 0.039 (NS)	*p* = 0.781	*p* = 0.296
LSOT5		X	*p* = 0.054	*p* = 0.0064 (NS)
Normal_MV			X	*p* = 0.171
LSOT5_MV				X

ML1V (Friedman Test, *p* < 0.001)				
	LSOT1	LSOT5	LSOT1_MV	LSOT5_MV
Normal	X	**p < 0.0001 (S)**	**p < 0.0001 (S)**	**p < 0.0001 (S)**
LSOT5		X	*p* = 0.156	*p* = 0.019 (NS)
Normal_MV			X	*p* = 0.280
LSOT5_MV				X

ML2 (Mixed ANOVA, interaction: *p* < 0.001)				
	LSOT1	LSOT5	LSOT1_MV	LSOT5_MV
Normal	X	*p* = 0.042 (NS)	*p* = 0.669	*p* = 0.101
LSOT5		X	*p* = 0.075	*p* = 0.0018 (NS)
Normal_MV			X	*p* = 0.005 (NS)
LSOT5_MV				X

ML2V (Friedman Test, *p* < 0.001)				
	Normal	LSOT5	Normal_MV	LSOT5_MV
Normal	X	**p < 0.0001 (S)**	**p = 0.0006 (S)**	**p < 0.0001 (S)**
LSOT5		X	*p* = 0.690	*p* = 0.006 (NS)
Normal_MV			X	*p* = 0.027
LSOT5_MV				X

Bold represents *p* < 0.0007.

### 3.4 The size effect

The partial eta squared values were 0.283 for AP1, 0.102 for ML1, and 0.187 for ML2. The Kendalls W values were 0.807 for V1, 0.455 for V1V, 0.584 for V2, 0.7 for V2V, 0.645 for AP1V, 0.384 for AP2, 0.717 for AP2V, 0.385 for ML1V, and 0.39 for ML2V. These values supported that the effect size of this study wad from medium to large effect.

## 4 Discussions

Studying the ground reaction force (GRF) while walking under vestibular-demanding conditions (LSOT1) with or without mastoid vibrations (MV) aimed to elucidate how humans redistribute forces compared to normal walking (LSOT1) with or without MV. This study confirmed the hypotheses that 1) walking in LSOT5 increased the GRF variabilities in V and AP directions, 2) walking with MV decreased the GRFs in V and AP directions; and 3) walking in LSOT5 with MV increased the GRF variability most compared to walking in LSOT1 without MV, indicating that LSOT5 can be used to identify the deteriorations in vestibular system. The results rejected our hypotheses that 1) walking in LSOT five did not affect the GRFs compared to walking in LSOT1 with/without MV; and 2) there was no effect of LSOT conditions or the effect of MV on GRFs in ML directions.

### 4.1 Walking in the vestibular-demanding task (LSOT5) necessitated adjustments in GRF from one stance cycle to another, irrespective of whether mastoid vibrations were administered

It has been demonstrated that various forms of vestibular stimulation significantly influence the margin of stability variability in both the anterior-posterior and medial-lateral directions in young adults (Lu et al., 2022). Additionally, simply walking blindfolded has been shown to increase step length variability (Bauby and Kuo, 2000), while walking on an oscillating surface increases trunk variability in both the anterior-posterior and medial-lateral directions (McAndrew et al., 2010). Moreover, studies have indicated that walking in LSOT5 increases net center of pressure sway variability compared to walking in LSOT1 (Chien et al., 2014). These findings collectively suggest that less reliable sensory systems may result in greater variability. The present study observed significant increases in GRF variabilities in the V and AP directions when walking in LSOT5 compared to LSOT1 regardless of whether the MV was administrated (V2: 2.23 (LSOT1) vs. 5.36 (LSOT5), an increase in 140.35%; AP2: 10.89 (LSOT1) vs. 17.03 (LSOT5), an increase in 56.38%) or not (V2: 2.07 (LSOT1) vs. 4.33 (LSOT5), an increase in 109.17%; AP2: 8.21 (LSOT1) vs. 15.32 (LSOT5), an increases in 86.60%). This observation may be explained by the concept of the internal model (Merfeld et al., 1999), which refers to the brain’s ability to store information about the external environment related to the individual’s surroundings. According to Ito (2008), the internal model of stability control consists of several major components, including the instructor (prefrontal cortex), the controller (motor cortex), the controlled object (body parts), the sensory systems (visual, somatosensory, vestibular systems), and the internal model itself (forward or inverse model). During walking in LSOT5, where participants were navigating an unfamiliar condition, the instructor (prefrontal cortex) initially receives environmental information primarily from the vestibular system, with a lesser reliance on the visual and somatosensory systems. Subsequently, the instructor provides instructions to the controller (motor cortex), sending motor commands to the controlled objects to maintain stability. Concurrently, the controller sends a signal back to the internal model to compare the actual body position with the predicted position based on the forward model. If disparities between the predicted and actual body positions are detected, the internal model may correct these differences and transmit the corrections back to the instructor. This iterative process of correction is likely repeated to counteract unpredictable sensory conflicts from the visual and somatosensory systems during walking in LSOT5. Consequently, these continual corrections may lead to stride-to-stride adjustments, resulting in greater GRF variability in LSOT5 compared to LSOT1.

It was worth mentioning that the significant differences in GRFs between LSOT one and LSOT five regardless of whether MV was administrated or not were not found in this study. It might be the experimental design that the mean of treadmill was 99.3% of the preferred walking speed in LSOT5, which was very close to the preferred walking speed in LSOT1. It has been shown that the amplitudes of GRFs are highly correlated to the walking speed (Nilsson and Thorstensson, 1989); therefore, it may be a possibility that the GRFs was averaged out as a result of step-by-step adjustments (greater GRF variabilities) associated with slowed-down and sped-up treadmill settings in LSOT5.

### 4.2 Walking with bilateral mastoid vibrations reduced the peaks of GRF

First and foremost, mastoid vibration (MV) at a stimulation frequency of 100 Hz has been utilized for decades to assess various types of vestibular disorders, including unilateral vestibular loss (Lucke, 1973), unilateral vestibular lesions (Dumas et al., 2007), partial unilateral vestibular lesions (Dumas et al., 2011), vestibular neuritis (Karlberg et al., 2003), and superior semicircular canal dehiscence (Dumas et al., 2014). The aforementioned studies have employed vibration-induced nystagmus, an abnormal eye movement, to gauge the efficacy of MV in diagnosing these diverse forms of vestibular disorders. For example, in individuals with partial or total unilateral vestibular lesions, stimulating both sides of the mastoid process results in vibration-induced nystagmus shifting away from the affected side (Lucke, 1973; Yagi and Ohyama, 1996; Hamann and Schuster, 1999; Karlberg et al., 2003; Dumas et al., 2007). Conversely, in patients with superior semicircular canal dehiscence (Dumas et al., 2014), vibration-induced nystagmus shift toward the side of the lesion when MV is applied. Interestingly, when individuals with bilateral areflexia and symmetrical hypofunction stimulate both sides of the mastoid process, no changes in vibration-induced nystagmus are observed (Dumas et al., 2011; Dumas et al., 2014). Moreover, Kavounoudias et al. (1999) applied vibration to the mastoid process and observed that the body moved toward the opposite direction from where the vibration was applied unilaterally. When two skull vibrators were positioned perpendicular to each other, the body moved diagonally, and when placed on both sides of the head in similar locations, the body moved forward. Kavounoudias et al. (1999) suggested that vibrations induced vestibular-proprioceptive processing and generated the vestibular illusion. For instance, when vibration activated the dorsal neck muscles, a proprioceptive signal suggested that the head was inclined forward relative to the trunk, while the vestibular signal indicated that the head remained straight. Consequently, the forward-directed postural sway induced by this dorsal neck vibration is likely a compensatory response aimed at restoring the body to an upright position. Similar observations were found in Chien et al., 2016; Chien et al., 2017, where walking in LSOT5 with bilateral MV significantly increased the degree of freedom of net center pressure movement in the AP direction compared to walking in LSOT5 without MV in both young and older adults. In the present study, applying bilateral mastoid vibrations might have generated the aforementioned vestibular illusion, causing the body to continuously move forward during treadmill walking and further increase the cadence (Dakin et al., 2013; Sun et al., 2023). Increased cadence resulted in reduced GRF peaks (Musgjerd et al., 2021). Thus, this might be why the reductions in GRFs with MV than without MV in V (V1: 1.25 of body weight (LSOT1) vs. 1.17 of body weight (LSOT1MV), a decrease in 6.4%; V1: 1.28 of body weight (LSOT5) vs. 1.17 of body weight (LSOT5MV), a decrease in 8.59%; V2: 1.29 of body weight (LSOT1) vs. 1.21 of body weight (LSOT1MV), a decrease in 6.2%; V2: 1.31 of body weight (LSOT5) vs. 1.2 of body weight (LSOT5MV), a decrease in 8.39%) and AP directions (AP1: 0.35 of body weight (LSOT5) vs. 0.26 of body weight (LSOT5MV), a decrease in 25.71%; AP2: 0.34 of body weight (LSOT5) vs. 0.28 of body weight (LSOT5MV), a decrease in 17.64%) were observed in the present study.

### 4.3 Walking in LSOT5 with bilateral mastoid vibration posed a greater challenge

In the present study, the lowest amplitudes of GRFs were observed in V1 (1.17 of body weight), V2 (1.2 of body weight), AP1 (0.26 of body weight), ML1 (0.20 of body weight), and ML2 (0.21 of body weight), while the highest variabilities were found in V2V (5.37), AP1V (18.58), AP2V (17.03), ML1V (14.66), and ML2V (14.17) when walking in LSOT5 with mastoid vibrations compared to walking in LSOT1 without mastoid vibrations (V1: 1.24, V2: 1.29, AP1: 0.31, ML1: 0.22, ML2: 0.23 of body weight; V2V: 2.07, AP1V: 10.19, AP2V: 8.21, ML1V: 9.44, ML2V: 8.67). It was noted that greater negative values indicated greater GRF in the AP. These changes are likely attributed to walking in a vestibular-demanding environment and the vestibular system was disrupted or deteriorated. This observation aligned with Wood et al.’s (2015), which applied SOT5 to measure vestibular function in 37 astronauts upon returning to Earth’s surface, revealing an 82.4% increase in sway post-spaceflight compared to pre-flight, indicating significant vestibular deterioration in microgravity. Mulavara et al. (2010) designed a functional mobility test to assess locomotor function post-spaceflight, including passing through vertical pylons, a gate, and a couple of obstacles. The surface was built with a compliant foam surface to “make the support surface unreliable,” similar to LSOT5’s design, inducing sudden acceleration/deceleration in the AP direction. They found that astronauts who spent 163–195 days in the International Space Station took double the time to complete this functional mobility test upon landing, suggesting sensory system deterioration, particularly in the vestibular function. The findings of our study (the significant differences in GRF between with and without MV when walking in LSOT5) have dual implications: firstly, walking under LSOT5 could potentially differentiate vestibular status across various scenarios using GRF measure, such as pre-and post-spaceflight or pre- and post-landing in different gravity levels, and may indicate the duration required for full vestibular system recovery after returning from space. Secondly, training in walking under LSOT5 with mastoid vibration could enhance locomotor adaptation capabilities in sensory-conflicted situations. Previous research has demonstrated that treadmill-induced perturbation training improves balance and reduces fall risk in older adults (Kurz et al., 2016), suggesting that incorporating LSOT5 with mastoid vibration into future sensorimotor training protocols may enhance sensory integration capabilities in astronauts navigating unpredictable environments, such as quicksand on a dark night under varying gravity levels.

### 4.4 Why did walking with/without mastoid vibration have no effect on the mean GRFs but increase the GRF variabilities in the ML directions?

Unexpectedly, no significant differences in GRFs in the ML direction was observed when walking with or without MV. Bauby and Kuo’s study (2000) suggested that active control in the ML direction was heavily necessary when walking blindfolded (actively controlled) by measuring their step width variability. McGeer (1990) demonstrated that a bipedal robot without any control mechanism could mimic human-like gait in the AP direction while walking downhill. However, this robot was prone to falling sideways due to the lack of a control mechanism, supporting the active control hypothesis. Additionally, research has indicated that walking on sinusoidal surface oscillations in the anterior-posterior (AP) direction impacts the margin of stability in the medial-lateral (ML) directions (McAndrew-Young et al., 2012). A study even demonstrated that walking in vestibular-demanding tasks, such as narrow walking with galvanic vestibular stimulation, requires active control in the ML direction, as evidenced by increased GRF peak amplitudes in the ML direction and muscle activations in the erector spinae (Magnani et al., 2021). These findings contradicted the results of the present study, which found that treadmill-induced perturbations in the AP direction did not affect the GRF peaks in the ML directions. A possible rationale to explain the no significant observations on mean GRFs in the present study was that the changes in GRFs in the ML direction were averaged out due to the mean treadmill speed was similar within the LSOT conditions (LSOT1: 100% of PWS, and LSOT5: 99.29% of PWS) and between the groups. Research has demonstrated a negative linear correlation between step width and walking speed when walking at 80%–120% of preferred walking speed (Brinkerhoff et al., 2023). Therefore, in LSOT5, after averaging the walking speed over 17 types of treadmill-induced perturbations (mixed acceleration and deceleration of the treadmill speed), the changes in gait characteristics, which influenced the GRF, in the ML direction should be similar to those observed in LSOT1, regardless of whether MV was administered or not. Hu and Chien (2021) confirmed this hypothesis, demonstrating that the step width while walking in the LSOT5 (12.35 cm) were similar to those observed in the LSOT1 (12.18 cm). In contract, a significantly GRF variability in the ML direction and step width variability were noted in the previous (Hu and Chien, 2021) and current study. This GRF variability, step-to-step adjustment, can be attributed to the foot placement strategy, which regulated the location of foot placement at the moment of heel contact as needed. By effectively positioning the center of pressure mediolaterally in relation to the center of mass, the GRF generates a moment that helps prevent falls (van Leeuwen et al., 2020). Furthermore, the step-by-step foot placement was influenced by the activity of the hip abductor and adductor muscles during steady-state walking (hip strategy, van Leeuwen et al., 2020). It has been shown that walking in LSOT5 necessitated step-to-step adjustments in the ML direction compared to walking in LSOT1, as evidenced by the 95% confidence ellipse area of foot placement distribution (Hu and Chien, 2021). Specifically, their investigation revealed that the length of the short axis of the ellipse in the ML direction increased by 25.65%, from 33.96 mm (LSOT1) to 42.67 mm (LSOT5), while the length of the long axis (AP direction) increased by 55.47%, from 64.34 mm to 100.05 mm in healthy young adults. In the current study, the greater GRF variability necessitated larger step-to-step adjustments when walking in LSOT5 without MV, in LSOT1 with MV, or in LSOT5 with MV compared to walking in LSOT1 without MV. This finding indicated that step-to-step adjustments were essential, suggesting that whenever engaging in vestibular-demanding tasks or under conditions that disrupt the vestibular system, active control in a step-to-step manner in the ML direction was highly demanded. Although the hip strategy could not be measured in this study due to the absence of a motion capture system, it was likely speculated that the hip strategy would be utilized while walking in LSOT5, regardless of whether MV was administered. Furthermore, when the bilateral MV was applied and walking in LSOT5, these participants may have had no choice but to primarily use the hip strategy to actively adjust their step-to-step foot placement.

### 4.5 Conclusions and limitations

Several limitations warrant consideration for future studies.

• This study focused exclusively on young adults, and future research should encompass astronauts both pre- and post-spaceflight to enhance applicability.
• Walking speed was not controlled, as imposing a fixed speed among participants might alter participants’ natural gait patterns. Consequently, variations in stance cycles across participants and trials could potentially influence the means and variability of GRF peaks. Thus, the coefficient of variation was utilized as the variability measure to mitigate this limitation.
• The absence of joint angle, head, and trunk movement tracking may have limited the ability to directly elucidate the effects of visual manipulations and treadmill-induced perturbations on the vestibular system and the applied strategies, such as hip and ankle strategies during walking. Future investigations should include joint angle, head and trunk movements tracking to address this limitation.
• The impulse of GRFs in each direction comprises two components: GRFs and time duration. Hence, the duration of each stance phase may vary among participants. Additionally, the number of stance phases may differ between participants and even within the same individual across different conditions. Consequently, the analysis of GRF impulse parameters was excluded at the present time.
• The impact of spacesuits on gait patterns should also be considered in future studies.

In summary, this pioneering study underscores the distinct responses of ground reaction force and its variabilities to different sensory challenges during walking. This paradigm may shed light on potentially assessing vestibular function during walking and aiding sensorimotor adaptation in future space missions.

## Data Availability

The original contributions presented in the study are included in the article/Supplementary Material, further inquiries can be directed to the corresponding author.

## Abbreviations

SOT, Sensory organization test; LSOT, locomotor sensory organization test; V1, the mean of the first peaks of GRF in the vertical direction; V2, the mean of the second peaks of GRF in the vertical direction; AP1, the mean of the first peaks of GRF in the anterior-posterior direction; AP2, the mean of the second peaks of GRF in the anterior-posterior direction; ML1, the mean of the first peaks of GRF in the medial-lateral direction; ML2, the mean of the second peaks of GRF in the medial-lateral direction; V1V, the variability of the first peaks of GRF in the vertical direction; V2V, the variability of the second peaks of GRF in the vertical direction.; AP1V, the variability of the first peaks of GRF in the anterior-posterior direction; AP2V, the variability of the second peaks of GRF in the anterior-posterior direction; ML1V, the variability of the first peaks of GRF in the medial-lateral direction; ML2V, the variability of the second peaks of GRF in the medial-lateral direction.
